# Research on Coverage Optimization in a WSN Based on an Improved COOT Bird Algorithm

**DOI:** 10.3390/s22093383

**Published:** 2022-04-28

**Authors:** Yihui Huang, Jing Zhang, Wei Wei, Tao Qin, Yuancheng Fan, Xuemei Luo, Jing Yang

**Affiliations:** 1 Electrical Engineering College, Guizhou University, Guiyang 550025, China; gs.yhhuang20@gzu.edu.cn (Y.H.); zhangjing@gzu.edu.cn (J.Z.); tqin@gzu.edu.cn (T.Q.); xmluo1@126.com (X.L.); 2 Power China Guizhou Electric Power Engineering Co., Ltd., Guiyang 550025, China; weiwei-gzy@powerchina.cn; 3 Power China Guizhou Engineering Co., Ltd., Guiyang 550001, China; fanyc-gzgc@powerchina.cn; 4 Key Laboratory of Advanced Manufacturing Technology of the Ministry of Education, Guizhou University, Guiyang 550025, China

**Keywords:** wireless sensor networks, COOT bird optimization algorithm, chaotic tent map, Lévy flight, opposition-based learning, coverage optimization

## Abstract

To address the problems of uneven distribution and low coverage of wireless sensor network (WSN) nodes in random deployment, a node coverage optimization strategy with an improved COOT bird algorithm (COOTCLCO) is proposed. Firstly, the chaotic tent map is used to initialize the population, increase the diversity of the population, and lay the foundation for the global search for the optimal solutions. Secondly, the Lévy flight strategy is used to perturb the individual positions to improve the search range of the population. Thirdly, Cauchy mutation and an opposition-based learning strategy are fused to perturb the optimal solutions to generate new solutions and enhance the ability of the algorithm to jump out of the local optimum. Finally, the COOTCLCO algorithm is applied to WSN coverage optimization problems. Simulation results show that COOTCLCO has a faster convergence speed and better search accuracy than several other typical algorithms on 23 benchmark test functions; meanwhile, the coverage rate of the COOTCLCO algorithm is increased by 9.654%, 13.888%, 6.188%, 5.39%, 1.31%, and 2.012% compared to particle swarm optimization (PSO), butterfly optimization algorithm (BOA), seagull optimization algorithm (SOA), whale optimization algorithm (WOA), Harris hawks optimization (HHO), and bald eagle search (BES), respectively. This means that in terms of coverage optimization effect, COOTCLCO can obtain a higher coverage rate compared to these algorithms. The experimental results demonstrate that COOTCLCO can effectively improve the coverage rate of sensor nodes and improve the distribution of nodes in WSN coverage optimization problems.

## 1. Introduction

### 1.1. Background of Problem

Wireless sensor networks (WSNs) are composed of a large number of low-power sensor nodes with communication functions, and have been widely used in military, industrial, and agricultural control, urban management, biomedicine, environmental detection, disaster relief, and other fields [1,2]. The coverage problem is one of the most fundamental problems in wireless sensor networks, and coverage is an important indicator for evaluating coverage optimization strategies, which has a great impact on the quality of service of wireless sensor networks because it directly determines the monitoring capability of the target monitoring area in the wireless sensor network. Rational and effective deployment of sensor nodes not only minimizes the network cost, but also reduces energy consumption in the sensor power optimization problem [3,4,5,6]. In coverage applications of wireless sensor networks, in order to improve coverage efficiency, all aim to deploy a minimum number of sensor nodes to monitor a specific target area of interest. Generally, sensor nodes are randomly deployed in the target monitoring area, resulting in uneven distribution of sensor nodes and, thus, bringing low coverage. Therefore, it is of great importance to improve the coverage of wireless sensor networks in the monitoring area by deploying sensor nodes rationally and efficiently [7,8].

### 1.2. Related Works

The node deployment problems in wireless sensor networks can be solved by building an integer linear programming model and then using the methods for solving it. In general, the branch-and-bound method is commonly used to solve the integer programming problem [9,10,11]. For small-scale node deployment problems, integer linear programming methods can be used to solve them. However, for large-scale sensor node deployment problems, the rational and efficient deployment of wireless sensor nodes has been proven to be an NP-hard problem, and finding the optimal solution for such problems remains a challenge. In this background, scholars have proposed the use of metaheuristic algorithms as a solution. Metaheuristic algorithms are approximate optimization algorithms with solutions that escape from local optima, and are widely regarded as effective methods for solving high-dimensional optimization problems, as well as various complex engineering problems. Metaheuristic algorithms can find near-optimal solutions in a reasonable time using limited computational resources, providing a very effective approach to the coverage optimization problems for wireless sensor networks [12,13,14]. Metaheuristic algorithms are classified into four categories of algorithms: evolutionary concepts, animal behavior, physical phenomena, and human behavior [15,16,17]. The first category is based on evolutionary concepts, including genetic algorithms (GAs) [18], genetic programming (GP) [19], evolutionary programming (EP) [20], differential evolution (DE) [21], and biogeography-based optimizers (BBOs) [22]. The second category is based on animal behavior, such as the coot optimization algorithm (COOT) [17], particle swarm optimization (PSO) [23], grey wolf optimizer, GWO) [24], salp swarm algorithm (SSA) [25], butterfly optimization algorithm (BOA) [26], seagull optimization algorithm (SOA) [27], whale optimization algorithm (WOA) [28], Harris hawks optimization (HHO) [29], and bald eagle search (BES) [30]. The third category consists of algorithms based on physical phenomena in nature, such as simulated annealing (SA) [31], black hole algorithm (BH) [32], sine–cosine algorithm (SCA) [33], and ray optimization (RO) [34]. The fourth category is human-behavior-based algorithms, such as teaching–learning-based optimization (TLBO) [35], harmony search (HS) [36], exchange market algorithm (EMA) [37], imperialist competitive algorithm (ICA) [38], and political optimizer (PO) [39].

There are some studies on using metaheuristic algorithms for WSN coverage optimization problems. In [40], ZainEldin et al. proposed a dynamic deployment strategy based on IDDT-GA to maximize the area coverage rate with the minimum number of sensor nodes. However, the proposed algorithm tends to fall into local optima, which affects the optimization of coverage, and there are too few compared algorithms for it to be convincing enough. In [41], Zhang et al. proposed an SA-GWO algorithm for the problem of high aggregation and low coverage rate when sensor nodes are deployed randomly. Although a better coverage effect was achieved, the time complexity of the algorithm was high. In [42], Liu et al. used the ALO algorithm to address the problems of uneven node distribution and incomplete coverage in node deployment. However, the coverage optimization of this algorithm was not ideal, and there was still an uneven distribution of sensor nodes. In [43], Liu et al. proposed an EFWA algorithm to solve the dynamic deployment problem of mobile sensor networks. However, the algorithm would easily fall into a local optimum too early, resulting in a lower coverage rate. In [44], Liao et al. used the GSO algorithm to improve the coverage after random deployment. However, there were obvious coverage holes and node redundancy. In [45], Ozturk et al. used the ABC algorithm to address the dynamic deployment problem in mobile and fixed-sensor scenarios, but there were too few compared algorithms, and the optimized experimental results still had node redundancy. In [46], Zhu et al. proposed an improved hybrid strategy weed algorithm to solve coverage optimization problems of WSNs. However, the problem of coverage holes still existed. It can be seen that it is of value and significance to use metaheuristic algorithms or improved metaheuristic algorithms for node coverage optimization problems. At the same time, the common problems of metaheuristic algorithm, such as slow convergence speed and susceptibility to falling into local optima, will lead to poor coverage optimization effects, which is also the focus of improvement in this paper.

The COOT optimization algorithm [17]—a novel metaheuristic algorithm proposed by the Iranian scholar Iraj Naruei in 2021—has been rapidly applied in the field of engineering optimization. In [47], Memarzadeh et al. used the COOT algorithm to optimize the parameters of a wind power prediction model. In [48], Gouda et al. used the COOT optimization algorithm to test the performance of solar power generator units. In [49], Mahdy et al. combined the COOT optimization algorithm with an anti-windup approach as a way to improve the transient stability of a wave energy conversion system (WECS). In [50], Houssein et al. proposed an improved COOT optimization algorithm for identifying the optimal lithium-ion (Li-ion) battery model parameters. In [51], Alqahtani et al. proposed a COOT-CMO algorithm for selecting the appropriate optimal candidate terms in the automatic query expansion process.

Although the COOT algorithm has been successfully applied and solved some engineering optimization problems, it also has problems, such as sluggish convergence speed and susceptibility to falling into local optima, the same as other metaheuristic algorithms. Therefore, in order to improve the flaws in the original algorithm, four effective strategies—namely, chaotic tent map, Lévy flight, Cauchy mutation, and opposition-based learning—are introduced to the algorithm to propose an improved COOT optimization algorithm, named COOTCLCO.

### 1.3. Contributions

The main contributions of this paper are as follows:

1. A new and improved algorithm named COOTCLCO, based on the COOT bird algorithm, is proposed.
2. Population diversity is improved by introducing the chaotic tent map to initialize populations. Expanding the search range of the populations by introducing the Lévy flight strategy, the capability of the algorithm to jump out of the local optimum is enhanced by introducing the Cauchy mutation and the opposition-based learning strategy.
3. The optimization capability of the proposed algorithm is tested on unimodal, multimodal, and fixed-dimension multimodal benchmark test functions.
4. The proposed algorithm is compared with seven metaheuristic algorithms in numerical analysis and convergence curves for the performance of finding the best optimal value.
5. An integer linear programming model is used to describe the coverage optimization problem of wireless sensor networks, and the proposed algorithm is used to solve this optimization problem. The proposed algorithm is compared with six metaheuristic algorithms in the coverage optimization problem.

### 1.4. Notations

The following Table 1 illustrates all of the notations that appear in this paper:

### 1.5. Organization

The remainder of this paper is organized as follows: Section 2 introduces the node coverage model of WSN. Section 3 describes the basic principles of the COOT optimization algorithm in detail. Section 4 details the improvement strategies of the COOT optimization algorithm. Section 5 introduces the coverage optimization strategy. Section 6 details the experimental design scheme, benchmark test function search performance comparison, coverage optimization performance comparison, and the practical application of COOTCLCO in addressing WSN coverage optimization problems. Section 7 gives a summary.

## 2. WSN Node Coverage Model

Suppose that *q* sensor nodes are randomly deployed in a two-dimensional WSN monitoring area with an area of *M* × *N* m^2^, where the set of nodes can be denoted as $S=\left\{S_{1},S_{2},\cdot \cdot \cdot S_{i},\cdot \cdot \cdot S_{q}\right\}$, and the coordinates of each node $S_{i}$ can be denoted as $\left(x_{i},y_{i}\right)$, where i=1,2,⋅⋅⋅q.

For a two-dimensional WSN monitoring area, the network model is as follows:

(1) Each sensor node is a homogeneous sensor; that is, it has the same parameters, structure, and communication capabilities.
(2) Each sensor node has sufficient energy, normal communication function, and timely access to data information.
(3) Each sensor node can move freely, and can update the location information in time.
(4) The sensing radius of each sensor node is $R_{s}$ and the communication radius is $R_{c}$, both in units of meters, and $R_{c}\geq 2R_{s}$.

The sensing range of a sensor node is a circular area, with the node itself as the center and the sensing radius $R_{s}$ as the radius. Assuming that there are *n* target monitoring points in this two-dimensional WSN monitoring area, the set of target monitoring points can be denoted as $T=\left\{T_{1},T_{2},\cdot \cdot \cdot T_{j},\cdot \cdot \cdot T_{n}\right\}$, and the location coordinates of each target point $T_{j}$ to be monitored are $\left(x_{j},y_{j}\right)$, where j=1,2,⋅⋅⋅n. If the distance between the target monitoring point $T_{j}$ and any of the sensor nodes is less than or equal to the sensing radius $R_{s}$, then it can be concluded that $T_{j}$ is covered by the sensor nodes. The Euclidean distance between sensor node $S_{i}$ and target monitoring point $T_{j}$ is defined as:

(1) $$d\left(S_{i},T_{j}\right)=\sqrt{{\left(x_{i}-x_{j}\right)}^{2}+{\left(y_{i}-y_{j}\right)}^{2}}$$

The node-sensing model in this paper is a Boolean sensing model; that is, when the sensing radius $R_{s}$ is greater than or equal to $d\left(S_{i},T_{j}\right)$, the probability that the target is monitored is 1; otherwise, the probability that the target is monitored is 0. If the probability that the target point $T_{j}$ to be monitored is covered by the sensor node $S_{i}$ be p, then

(2) $$p\left(S_{i},T_{j}\right)=\{\begin{array}{ll} 1 & R_{s}\geq d\left(S_{i},T_{j}\right)\\ 0 & R_{s}<d\left(S_{i},T_{j}\right) \end{array}$$

In this two-dimensional WSN monitoring area, the sensor nodes can work cooperatively with one another; that is, any target monitoring point can be covered by more than one sensor at the same time, so the probability that any monitoring target point $T_{j}$ is jointly sensed is:

(3) $$P\left(S,T_{j}\right)=1-\prod_{i=1}^{q}\left(1-p\left(S_{i},T_{j}\right)\right)$$

The coverage rate is defined as the rate of the coverage area of all sensor nodes in the monitoring area to the total area of the monitoring area; thus, the coverage rate of this 2D WSN monitoring area is:

(4) $$Cov=\frac{\sum_{j=1}^{n}P\left(S,T_{j}\right)}{M\times N}$$

Based on the above analysis, the node coverage optimization problem for wireless sensor networks can be described by the following integer linear programming model:

(5) $$Max\;Cov=\frac{\sum_{j=1}^{n}P\left(S,T_{j}\right)}{M\times N}$$

(6) $$s.t.\{\begin{array}{ll} \sum_{j=1}^{n}P\left(S,T_{j}\right)\geq 0 & 1\leq j\leq n\\ \sum_{j=1}^{n}P\left(S,T_{j}\right)\leq M\times N & 1\leq j\leq n\\ d\left(S_{i},T_{j}\right)\leq R_{s} & 1\leq i\leq q,1\leq j\leq n\; \end{array}$$

where Cov denotes the objective function for which the maximum coverage rate is required to be solved, $S_{i}$ denotes the *i*-th sensor node, $T_{j}$ denotes the *j*-th target point to be monitored, and *M* × *N* denotes the size of the monitoring area. The first constraint represents the probability constraint that any monitoring target point $T_{j}$ is jointly sensed. The second constraint indicates that the area covered by all sensor nodes in the monitoring area should be less than the total area of the monitoring area. The third constraint indicates that the Euclidean distance between the sensor node $S_{i}$ and the target monitoring point $T_{j}$ should be less than the sensing radius $R_{s}$ to effectively cover the target monitoring point.

When the size of the sensor nodes to be deployed is relatively large, it takes a lot of time to solve for the coverage problem using integer linear programming methods to obtain the optimal solution. To solve this puzzle effectively, a metaheuristic algorithm is appropriate, because metaheuristic algorithms can give satisfactory results in a tolerable time. Therefore, in this paper, an improved coot bird algorithm is proposed to solve the coverage optimization problem of wireless sensor networks.

## 3. COOT Optimization Algorithm

The principle of the COOT optimization algorithm is based on the different movement behaviors of coot flocks on the water surface. Coots are small waterbirds that have many different group behaviors on the water surface, with the ultimate goal of the behavior being to move toward food or a specific location. On the water surface, the coot group mainly has four different movement behaviors: random movement, chain movement, adjusting position according to the leader, and leader movement [17]. The process of implementing the COOT algorithm is composed of these four movement behaviors. The specific procedure of the algorithm is as follows [17]:

Initialize the population—random initialization of the population according to Equation (7):

(7) CootPos(i)=rand(1,d)×(ub−lb)+lb

where CootPos(i) is the position of the *i*-th coot, d is the number of variables or problem dimensions, ub is the upper bound of the search space, and lb is the lower bound of the search space. ub and lb are defined as follows:

(8) $$ub=[ub_{1},ub_{2},\cdot \cdot \cdot ub_{d}],lb=[lb_{1},lb_{2},\cdot \cdot \cdot lb_{d}]$$

After initializing the population, the position of the coot is updated according to the following four movement behaviors.

### 3.1. Random Movement

In this movement, a position Q is first initialized randomly using Equation (9):

(9) Q=rand(1,d)×(ub−lb)+lb

In order to avoid getting trapped in a local optimum, the position is updated according to Equation (10):

(10) $$CootPos(i)=CootPos(i)+A\times R_{2}\times (Q-CootPos(i))$$

where $R_{2}$ is a random number in the interval [0, 1], and A is determined from Equation (11):

(11) $$A=1-L\times (\frac{1}{Iter})$$

where Iter is the maximum number of iterations and L is the current number of iterations.

### 3.2. Chain Movement

The chain movement can be implemented by using the average position of the two coot birds, using Equation (12) to calculate the average position of the two coot birds:

(12) $$CootPos(i)=\frac{CootPos(i-1)+CootPos(i)}{2}$$

where CootPos(i−1) is the location of the second coot bird.

### 3.3. Adjusting Position According to the Leader

The coot bird updates its own position according to the position of the leader in the group; that is, the coot bird follower in each group moves towards the leader. The leader is selected according to Equation (13):

(13) K=1+(i MOD NL)

where K is the number of the leader, i is the number of the coot bird follower, and NL is the number of leaders.

In this movement, the coot bird updates its position according to Equation (14):

(14) $$CootPos(i)=LeaderPos(k)+2\times R_{1}\times \cos(2R\pi )\times (LeaderPos(k)-CootPos(i))$$

where CootPos(i) is the current position of the coot bird, LeaderPos(k) is the position of the selected leader, $R_{1}$ is a random number in the interval [0, 1], and R is a random number in the interval [−1, 1].

### 3.4. Leader Movement

In order to find the optimal position, the leader must jump from the existing local optimal position to the global optimal position, using Equation (15) to complete the leader position update:

(15) $$LeaderPos(i)=\{\begin{array}{ll} \begin{array}{l} B\times R_{3}\times \cos(2\pi R)\times \\ (gBest-LeaderPos(i))+gBest \end{array} & R_{4}<0.5\\ \begin{array}{l} B\times R_{3}\times \cos(2\pi R)\times \\ (gBest-LeaderPos(i))-gBest \end{array} & R_{4}\geq 0.5 \end{array}$$

where gBest is the best position that can be found, $R_{3}$ and $R_{4}$ are the random numbers between the interval [0, 1], and R is the random number between the interval [−1, 1]. B is determined from Equation (16):

(16) $$B=2-L\times (\frac{1}{Iter})$$

## 4. Improved COOT Optimization Algorithm

The basic COOT algorithm uses random initialization in the initialization process, which reduces the diversity of the initial population which, in turn, affects the performance of the algorithm. Due to the limitation of the search principle of the COOT algorithm, as the number of iterations increases, the individuals in the coot population gradually move closer to the leader with better fitness in the population, and if the leader cannot jump out of the local optimum in time, the whole population will easily fall into the state of local optimum, which reduces the algorithm’s search accuracy. Therefore, this paper proposes an improved COOT optimization algorithm: in the initial stage of the algorithm, a chaotic tent map is added to improve the diversity of the population and lay the foundation for improving the global search ability; subsequently, during the iterative process, the Lévy flight strategy is used to perturb the location of coot individuals to improve the search range of the population and reduce the phenomenon of falling into a local optimum; finally, at the optimal solution location, the Cauchy mutation and the opposition-based learning strategy are fused to perturb the mutation and generate a new solution to further enhance the capability of the algorithm to jump out of the local optimum, while improving the search accuracy of the algorithm.

### 4.1. Chaotic Tent Map Initializes the Population

Chaotic maps have the characteristics of ergodicity, randomness, and orderliness. Using chaotic variables for optimization searching can improve the diversity of populations and enable the algorithm to jump out of the local optimum, while improving the global search capability. The most common chaotic maps are tent maps, logistic maps, etc. Shan [52] demonstrated that tent maps have a faster search speed in combination with search algorithms compared to logistic maps. Li [53] also demonstrated the effectiveness of tent maps on a swarm intelligence algorithm to enhance population diversity. Therefore, in this paper, a tent map was selected to initialize the population. The expression for the chaotic tent map is defined as follows:

(17) $$z_{k+1}=\{\begin{array}{ll} \frac{z_{k}}{\alpha } & 0<z_{k}\leq \alpha \\ \frac{1-z_{k}}{1-\alpha } & \alpha <z_{k}\leq 1 \end{array}$$

The chaotic tent map is more effective when α is taken as 0.5, and the distribution of the sequence is more uniform at this time.

Therefore, the expression of the chaotic tent map in this paper is:

(18) $$z_{k+1}=\{\begin{array}{ll} 2z_{k} & 0<z_{k}\leq 0.5\\ 2(1-z_{k}) & 0.5<z_{k}\leq 1 \end{array}$$

**Figure 1:**
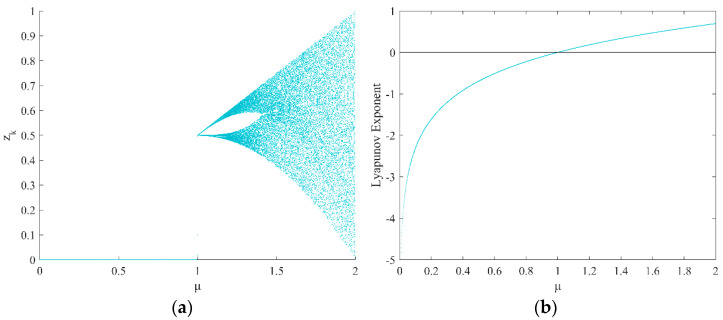
Chaotic tent map: (**a**) chaotic tent map bifurcation diagram; (**b**) Lyapunov exponential curve.

It can also be abbreviated as [54]:

(19) $$z_{k+1}=\mu \min\left\{z_{k},1-z_{k}\right\}$$

where μ is the parameter that controls the chaotic tent map, and here μ is taken as 2. According to the value of the parameter μ, the bifurcation diagram and Lyapunov exponential curve of the chaotic tent map were drawn, as shown in Figure 1. From Figure 1a, it can be seen that the chaotic tent map starts to bifurcate when the control parameter μ>1, and produces an approximately uniformly distributed chaotic sequence when μ=2; as can be seen from Figure 1b, when μ>1, that is, the Lyapunov exponent is greater than 0—it indicates that the chaotic tent map shows chaotic phenomena.

### 4.2. Lévy Flight Strategy

**Figure 2:**
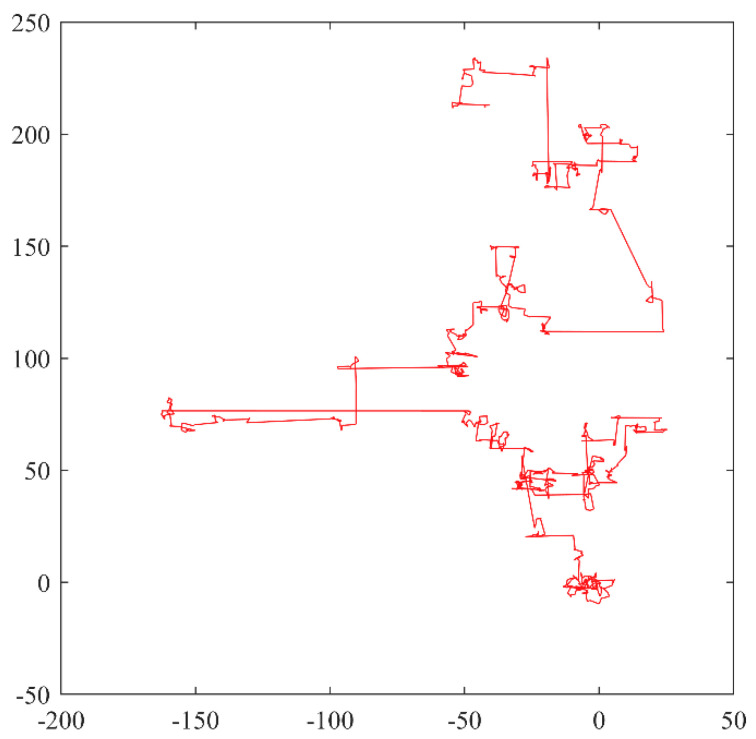
Lévy flight simulation diagram.

Lévy flight is a type of search for random walks obeying the Lévy distribution, characterized by the occurrence of long-range jumps as a class of non-Gaussian stochastic processes with Markovian properties, whose principle is derived from a probability distribution proposed by the French mathematician Paul Lévy [55]. The simulation of Lévy flight is shown in Figure 2.

Lévy flight involves a large jump in the search process, for which this paper introduces the Lévy flight mechanism to perturb the position update formula of the COOT algorithm. This can effectively boost the diversity of the population, expand the search range, improve the search capability of a single coot bird, and make the algorithm more easily jump out of the local optimum. Lévy flight can be described by the following mathematical Equation [56]:

(20) $$Levy\left(s,\gamma ,\mu \right)\{\begin{array}{ll} \sqrt{\frac{\gamma }{2\pi }}\exp\left[-\frac{\gamma }{2\left(s-u\right)}\right]\frac{1}{{\left(s-u\right)}^{\frac{3}{2}}} & 0<\mu <s<\infty ,\;\gamma \text{>}0\\ 0 & s\leq 0 \end{array}$$

where μ is the displacement parameter and γ is the scale parameter.

After Lévy flight is introduced, the position update Equation is [57]:

(21) $$x_{i}{}^{t+1}=x_{i}{}^{t}+\alpha ⊕Levy(\lambda )$$

where $x_{i}{}^{t+1}$ denotes the position after the Lévy flight perturbation; $x_{i}{}^{t}$ denotes the current position α denotes the step control factor; ⊕ denotes the dot product; and Levy(λ) denotes the random search path, indicating that it obeys the Lévy distribution with parameter λ [57]:

(22) $$Levy(\lambda )\sim u=t^{-\lambda },\;1\text{<}\lambda \leq 3$$

Computation of the Lévy flight random search path using the Mantegna algorithm is as follows [58]:

(23) $$s=\frac{\mu }{{\left|v\right|}^{\frac{1}{\beta }}},\;0\text{<}\beta <2$$

where β=λ−1, and β usually takes a value of 1.5. μ and v both obey a normal distribution [59]:

(24) $$\{\begin{array}{c} \mu \sim N(0,\delta_{u}{}^{2})\\ v\sim N(0,\delta_{v}{}^{2}) \end{array}$$

(25) $$\{\begin{array}{l} \begin{array}{l} \delta_{u}={\left[\frac{\Gamma (1+\beta )\times \sin(\frac{\pi \beta }{2})}{\Gamma (\frac{1+\beta }{2})\times \beta \times 2^{\frac{\beta -1}{2}}}\right]}^{\frac{1}{\beta }}\\ \delta_{v}=1 \end{array} \end{array}$$

Now, using the position update idea of Equation (21), the position update Equation of Equations (10), (12) and (14) are improved to obtain the following new position update Equations:

(26) $$CootPos(i)=CootPos(i)+A\times R_{2}\times (Q-CootPos(i))\times Levy$$

(27) $$CootPos(i)=\frac{CootPos(i-1)+CootPos(i)}{2}\times Levy$$

(28) $$\begin{array}{l} CootPos(i)=LeaderPos(k)+2\times R_{1}\times \cos(2R\pi )\times \\ \;(LeaderPos(k)-CootPos(i))\times Levy \end{array}$$

### 4.3. Fusing Cauchy Mutation and Opposition-Based Learning

Opposition-based learning [60] was proposed by Tizhoosh in 2005, and its primary idea is to take the current solution to a problem and then find its corresponding reverse solution through the opposition-based learning mechanism, and evaluate the original solution and the reverse solution to finally retain the optimal solution.

If x∈[a,b], then the distance from x to a is |x−a|, then the opposition-based learning number $x^{\prime }$ of x can be defined as follows [60]:

(29) $$x^{\prime }=a+b-x$$

**Figure 3:**
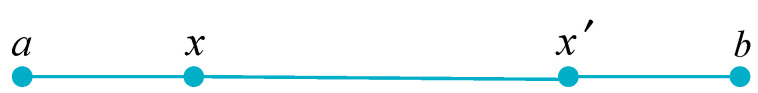
Relationship between arbitrary real numbers and their opposition-based learning numbers.

Then, the distance from $x^{\prime }$ to b is $\left|b-x^{\prime }\right|=\left|b-(a+b-x)\right|=\left|x-a\right|$, and these two distances are equal. The relationship between any real number and its opposition-based learning number is shown in Figure 3.

If $x=(x_{1},x_{2},\cdot \cdot \cdot ,x_{n})$ is a point in *n*-dimensional space, where $x_{i}\in [a_{i},b_{i}]$, the corresponding reverse point is [60]:

(30) $$x^{\prime }=({x^{\prime }}_{1},{x^{\prime }}_{2},\cdot \cdot \cdot ,{x^{\prime }}_{n})$$

Its corresponding reverse solution is [60]:

(31) $${x^{\prime }}_{i}=a_{i}+b_{i}-x_{i}$$

The basic COOT algorithm needs to select the optimal position by calculating the fitness after each iteration, and if its position update formula is not perturbed, the algorithm is prone to falling into a local optimum. In the previous section, this paper introduced Lévy flight to perturb its position update formula, but to further enhance the algorithm’s ability to jump out of local optima, this subsection combines Cauchy mutation and the opposition-based learning strategy to perform position update for the movement of adjusting position according to the leader. This step of the movement is where the coot bird updates its own position according to the position of the leader in the group, and it is important to choose the best leader for this phase. Therefore, we find its corresponding reverse solution according to the chosen leader position, which can provide more opportunities to find potential optimal solutions, further boost the diversity of the population based on the Lévy flight perturbation, enhance the global search capability of the algorithm, and prevent the algorithm from falling into a local optimum. Now, the idea of opposition-based learning and the COOT algorithm are combined, and Equation (31) is used to improve Equation (14), which leads to the reverse solution of the leader position and the position update equation based on opposition-based learning:

(32) $${X^{\prime }}_{LeaderPos}(i)=ub+r⊕(lb-X_{LeaderPos}(i))$$

(33) $$\begin{array}{c} X_{GbestNew}(i+1)={X^{\prime }}_{LeaderPos}(i)+b_{1}⊕2\times R_{1}⊕\cos(2\pi R)⊕\\ \left({X^{\prime }}_{LeaderPos}(i)-X_{CootPos}(i)\right) \end{array}$$

where ${X^{\prime }}_{LeaderPos}(i)$ in Equation (32) is the inverse solution of the current leader position at the *i*-th iteration, ub and lb denote upper and lower bounds, r denotes the random number matrix, and ⊕ denotes the dot product. $X_{GbestNew}(i+1)$ in Equation (33) denotes the latest position of the *i* + 1-th iteration, $R_{1}$ is a random number between the interval [0, 1], R is a random number between the interval [−1, 1], $X_{CootPos}(i)$ denotes the position of the coot bird follower of the *i*-th iteration, and $b_{1}$ denotes the information exchange control parameter, which is calculated as follows [61]:

(34) $$b_{1}={\left(1-\frac{i}{max\_Iter}\right)}^{i}$$

The Cauchy distribution is one of the common continuous-type distributions in probability theory, and its one-dimensional probability density function expression can be defined as follows [62]:

(35) $$f(x)=\frac{1}{\pi }\frac{T}{\left(x^{2}+T^{2}\right)},\;-\infty <x<\infty ,T>0$$

where T denotes the control parameter. The corresponding distribution function is [62]:

(36) $$F(x)=\frac{1}{2}+\frac{1}{\pi }\arctan(\frac{x}{T})$$

For Equation (35), when *T*=1, the standard Cauchy distribution probability density function [62] is obtained as shown in Equation (37):

(37) $$f(x)=\frac{1}{\pi }\frac{1}{\left(x^{2}+1\right)},\;-\infty <x<\infty$$

**Figure 4:**
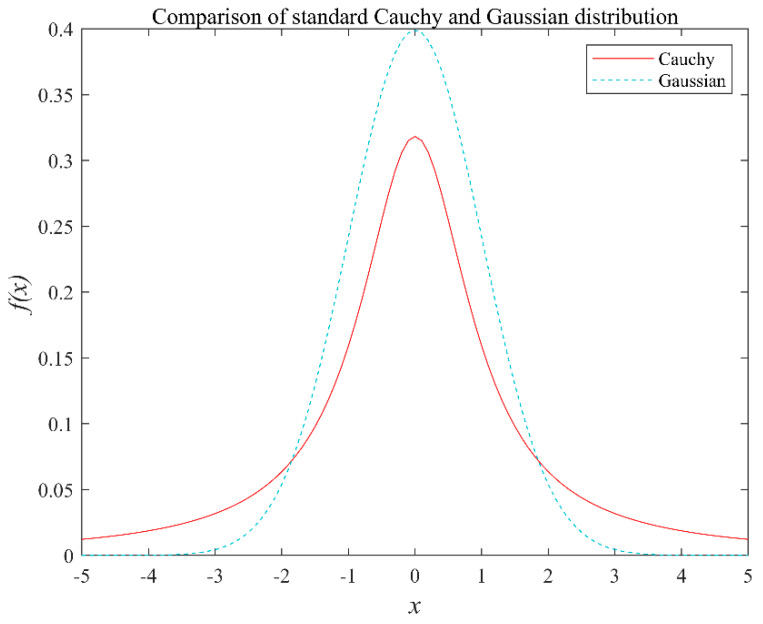
Probability density function curves for the standard Cauchy and Gaussian distributions.

A comparison of the probability density function curves of the standard Cauchy distribution and the Gaussian distribution [63] is shown in Figure 4. From the figure, it can be seen that the standard Cauchy distribution is similar to the standard Gaussian distribution, in that both of them are continuous probability distributions. However, the Cauchy distribution has a long and flat shape at both ends, approaches zero at a slower rate, and has a smaller peak near the origin compared to the Gaussian distribution. Therefore, the Cauchy distribution has a wider distribution range, and allows for greater mutation than the Gaussian distribution.

Introducing the Cauchy mutation into the position update formula of the COOT algorithm and exploiting the perturbation ability of the Cauchy mutation operator will further improve the diversity of the population, enhance the global search ability of the algorithm, and prevent the algorithm from falling into a local optimum. The new position update equation is generated using the Cauchy mutation as follows:

(38) $$X_{GbestNew}(i+1)=X_{CootPos}(i)+Cauchy(0,1)*X_{CootPos}(i)$$

where $X_{GbestNew}(i+1)$ denotes the latest position of the *i* + 1-th iteration after perturbation by the Cauchy mutation, $X_{CootPos}(i)$ denotes the position of the coot bird of the *i*-th iteration, and Cauchy(0,1) denotes the standard Cauchy distribution.

In order to enhance the performance of the algorithm for finding the best solution, the Cauchy mutation and the opposition-based learning strategy can be fused, and the selection probability *P_s_* for deciding which strategy to choose for the position update can be defined using a dynamic selection mechanism, so that both of them are executed alternately with a certain probability as follows [61]:

(39) $$P_{s}=-\exp{\left(1-\frac{i}{max\_Iter}\right)}^{20}+\eta$$

where η is the control parameter, and generally takes the value of 0.05 [61].

In this dynamic selection mechanism, if the random number $rand<P_{s}$, the position is updated using the opposition-based learning strategy; otherwise, the position is updated using the Cauchy mutation strategy.

### 4.4. Implementation Steps of COOTCLCO Algorithm

Step 1: Set the parameters of population size *N*, maximum number of iterations *max_Iter*, number of leaders $N_{Leader}$, number of followers $N_{coot}$, etc., and randomly initialize the positions of coot bird followers and leaders according to Equation (9).

Step 2: Enhance the diversity of the population by using the chaotic tent map in Equation (18).

Step 3: Update the position of the coot bird follower according to the Lévy flight perturbation strategy introduced by Equations (26)–(28).

Step 4: Update the position of the coot bird leader according to Equation (15).

Step 5: Calculate the fitness of the coot bird followers and leaders, and compare them to select the best fitness value.

Step 6: Select the Cauchy mutation or the opposition-based learning strategy according to Equation (39) to perturb the current optimal solution and generate a new solution.

Step 7: Determine whether the end condition is reached; if yes, proceed to the next step; otherwise, return to Step 3.

Step 8: The program ends and outputs the optimal fitness value and the best position.

### 4.5. COOTCLCO Algorithm Time Complexity Analysis

Suppose that the number of populations of the algorithm is *N*, the dimension of the search space is *D*, and the maximum number of iterations is *T*. Then, for the basic COOT algorithm, its time complexity is *O*(*NDT*). For the COOTCLCO algorithm, its time complexity is analyzed as follows:

(1) The time complexity of initializing the population using the chaotic tent map is *O*(*ND*). Thus, the required time complexity is *O*(*NDT*) + *O*(*ND*) = *O*(*NDT*) in the case of introducing only the chaotic tent map.
(2) The time complexity of perturbing the individual positions using the Lévy flight strategy is *O*(*ND*), and the time complexity of the algorithm is *O*(*NDT*) after T iterations. Thus, the required time complexity is *O*(*NDT*) + *O*(*NDT*) = *O*(*NDT*) when only the Lévy flight strategy is introduced.
(3) The time complexity of the algorithm is *O*(*NDT*) + *O*(*NDT*) after *T* iterations by fusing the Cauchy mutation and the opposition-based learning strategy and perturbing the optimal solution’s position. Thus, the required time complexity is *O*(*NDT*) + *O*(*NDT*) + *O*(*NDT*) = *O*(*NDT*) with the introduction of only the fused Cauchy mutation and the opposition-based learning strategy.

Therefore, after introducing the above three improvement strategies, the time complexity of the COOTCLCO algorithm is *O(COOTCLCO)* = *O*(*NDT*) + *O*(*NDT*) + *O*(*NDT*) = *O*(*NDT*). In summary, the time complexity of the COOTCLCO algorithm is the same as that of the COOT algorithm, thus showing that the improvement strategy proposed in this paper based on the COOT algorithm does not increase the time complexity of the algorithm.

## 5. Coverage Optimization Strategy

**Figure 5:**
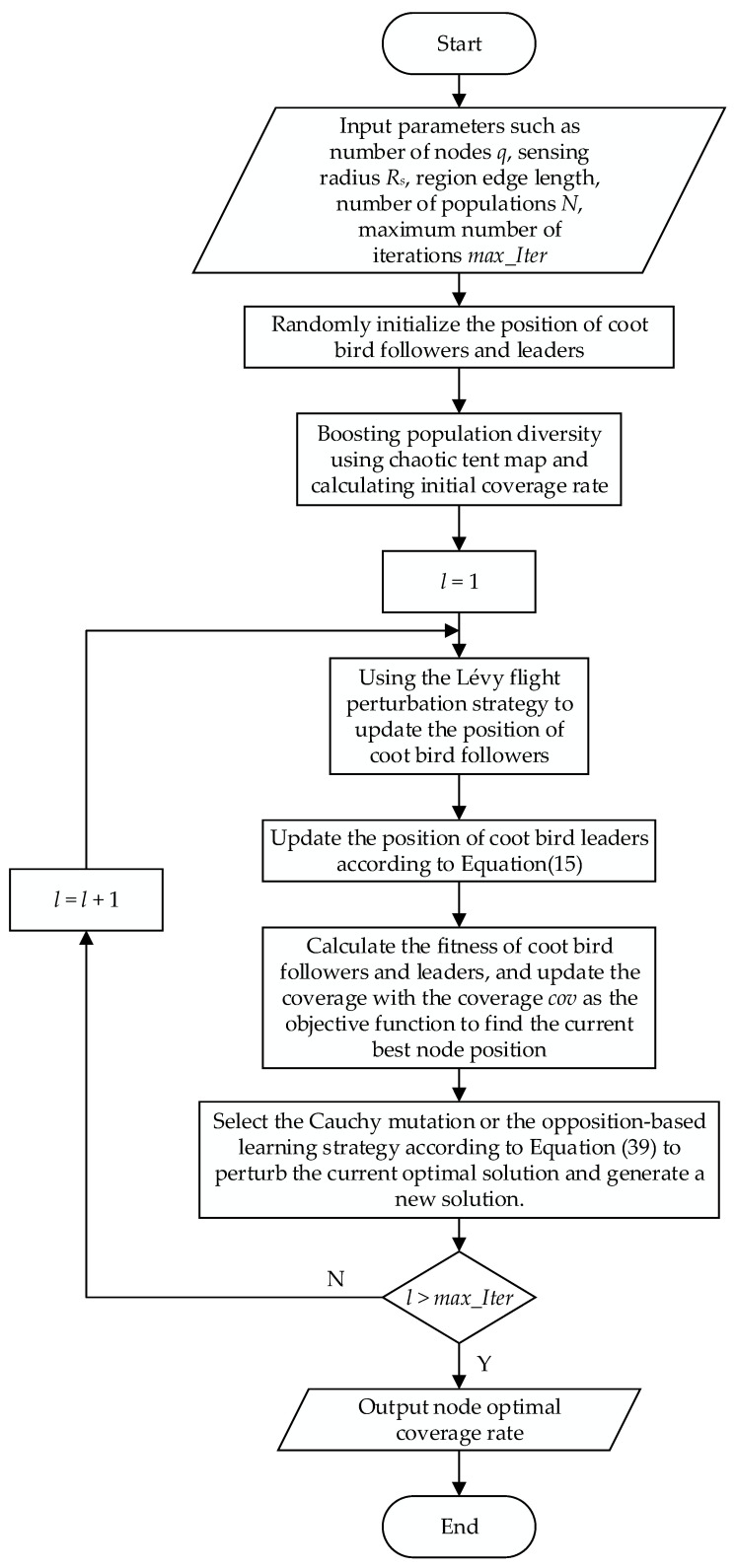
Flowchart of the COOTCLCO coverage optimization algorithm.

In this paper, the location-seeking process of nodes in the coverage optimization problem is abstracted as the process of making different movement behaviors of the coot bird group toward food or a specific location, and the optimal solution is the target location of each node deployed. The goal of WSN coverage optimization based on the COOTCLCO algorithm is to maximize the coverage of the target monitoring area by using a certain number of sensor nodes and optimizing the locations where they will be deployed. The flowchart of the coverage optimization algorithm is shown in Figure 5. Each coot bird individual in the algorithm represents a coverage distribution, and the specific algorithm steps are as follows:

Step 1: Input parameters such as the number of nodes *q*, perception radius *R*_s_, area of region *M* × *N*, etc., and randomly initialize the positions of the coot bird followers and leaders according to Equation (9).

Step 2: Boost the diversity of the population using the chaotic tent map in Equation (18), and calculate the initial coverage according to Equation (4).

Step 3: Update the position of the coot bird followers according to the Lévy flight perturbation strategy introduced by Equations (26)–(28).

Step 4: Update the position of the coot bird leaders according to Equation (15).

Step 5: Calculate the fitness of the coot bird followers and leaders, and update the coverage rate according to Equation (4), with the coverage rate *Cov* as the objective function, to find the current best node location.

Step 6: Select the Cauchy mutation or the opposition-based learning strategy according to Equation (39) to perturb the current optimal solution and generate a new solution.

Step 7: Determine whether the end condition is reached; if yes, proceed to the next step; otherwise, return to Step 3.

Step 8: The program ends and the node optimal coverage rate is output.

## 6. Simulation Experiments and Analysis

### 6.1. Experimental Design

In order to verify the optimization performance of COOTCLCO and its effectiveness in WSN node coverage optimization, two sets of comparative experiments were designed in this paper: (1) COOTCLCO was compared with the optimization performance of seven optimization algorithms, namely, COOT [17], PSO [23], GWO [24], SSA [25], BOA [26], SOA [27], and SCA [33]; (2) COOTCLCO was compared with six optimization algorithms—PSO, BOA, SOA, WOA [28], HHO [29], and BES [30]—on the WSN node coverage optimization problem.

In this paper, 23 benchmark functions were used to test the algorithm’s performance in finding the optimum, and these 23 benchmark functions can be divided into three categories. Table 2 lists 7 unimodal benchmark functions; Table 3 lists 6 multimodal benchmark functions with multiple local optimal solutions, and the number of local optimal solutions increases exponentially with the number of dimensions; and Table 4 lists 10 fixed-dimension multimodal benchmark functions.

Experimental simulation environment for this paper was as follows: Windows 10 OS, Intel Core i7-10750H CPU @2.60 GHz, NVIDIA GeForce GTX 1650Ti graphics card, SK Hynix 16 GB RAM, MATLAB 2019b simulation platform.

### 6.2. Performance Comparison on Benchmark Functions

In this section, the optimization ability of COOTCLCO is compared with that of COOT, PSO, GWO, SSA, BOA, SOA, and SCA. For all algorithms, the population size N=30 and the maximum number of iterations max_Iter=1500. In order to attenuate the chance of the experiment, each algorithm was run 30 times independently for the same test function, taking the average value and standard deviation of the experimental results for comparison, and the parameter settings of the comparison algorithms are detailed in Table 5. In order to test the difference between the COOTCLCO algorithm and the comparison algorithm, the Wilcoxon signed-rank test was used. At the significance level of 0.05, “+”, “≈”, and “−” indicate that the performance of COOTCLCO is superior to, similar to, or inferior to that of the comparison algorithm, respectively. Meanwhile, the average values derived from each algorithm under each test function were ranked. The experimental average value (avg), standard deviation (std), Wilcoxon signed-rank test results (W), and average value ranking (R) are presented in Table 6 and Table 7, and the best average value and standard deviation derived in each test function are shown in bold. Finally, the overall Wilcoxon signed-rank test results, the total average value ranking, and the overall algorithm ranking results are given in Table 8.

#### 6.2.1. Analysis of Numerical Results

The average value, standard deviation, Wilcoxon signed-rank test results, and average value ranking of the simulation experiments for the 8 optimization algorithms on the 23 benchmark test functions are given in Table 5, Table 6 and Table 7. The unimodal test function is suitable for evaluating the exploitation of the algorithms. In terms of average value and standard deviation, for the test functions F1, F2, and F5, GWO has the best performance, while COOTCLCO ranks as the second-best-performing algorithm. For F3 and F4, COOTCLCO is the best-performing optimizer, followed by COOT’s search ability, while SCA is the worst-performing optimization algorithm in these two test functions. For the test functions F6 and F7, COOTCLCO has an average performance in terms of the optimization ability, ranking fourth in the comparison of these eight algorithms tested. It is worth mentioning that in the test function F6, PSO exhibits the strongest optimization ability. Overall, among the unimodal test functions, COOTCLCO is the best overall performing algorithm in terms of optimization ability and stability, and is very effective and competitive with the other seven metaheuristics, while the test results show that COOTCLCO has a good exploitation capability.

Multimodal test functions and fixed-dimension test functions are suitable for assessing the exploration ability of the algorithms. Among the multimodal functions, for the test function F8, the optimization effect of the COOTCLCO algorithm is the best, while SSA and COOT are the second- and third-best-performing algorithms. For F9 and F10, COOTCLCO obtained the best results compared to the other algorithms; in F9, the second- and third-ranked test results were COOT and GWO, respectively; in F10, GWO and BOA were second only to COOTCLCO in terms of finding the best results. For F11, BOA had the best test results, while COOTCLCO followed closely. For F12, COOTCLCO outperformed the other algorithms in terms of average value and standard deviation. For F13, COOTCLCO’s test results ranked second, and it is worth mentioning that PSO performed the best in terms of average value and standard deviation results. Among the fixed-dimension functions, for F14, F20, and F21, COOTCLCO performed better than the other algorithms for both the average value and the standard deviation. For F15, the BOA test results were the best, and its standard deviation results prove that BOA has a strong stability in the test function F15, where the COOTCLCO test results rank second. For F16, F17, and F18, the eight algorithms can generally find the optimal value of the test function in terms of the average value of the optimal value; in terms of the standard deviation, COOTCLCO has the best stability; finally, COOTCLCO wins in the test results of F16, F17, and F18 in terms of stability. For F19, the PSO test results proved it to be the best performing algorithm, while COOTCLCO ranked as the second-best-performing algorithm. For F22 and F23, while COOTCLCO’s test results ranked only third and fourth, respectively, the best-performing GWO’s test results were only marginally stronger than COOTCLCO’s, as the two were very close in their optimization results.

All in all, for the multimodal and fixed-dimension test functions, COOTCLCO ranked first 10 times and second 4 times out of 16 test results, which is evidence that the COOTCLCO algorithm has an extremely strong exploration capability.

#### 6.2.2. Analysis of Convergence Curves

**Figure 6:**
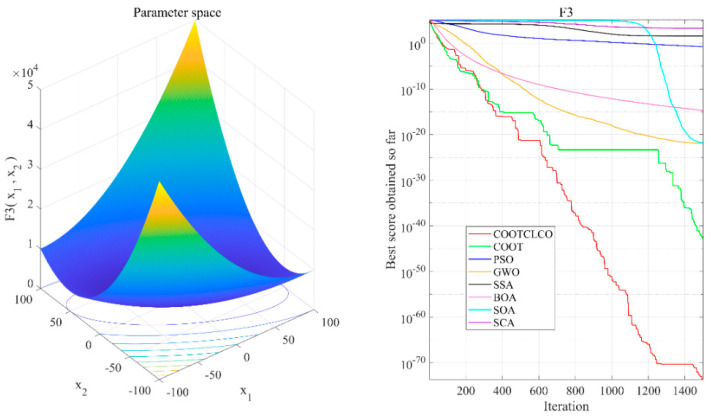
Test function F3 and comparison of convergence curves on F3.

**Figure 7:**
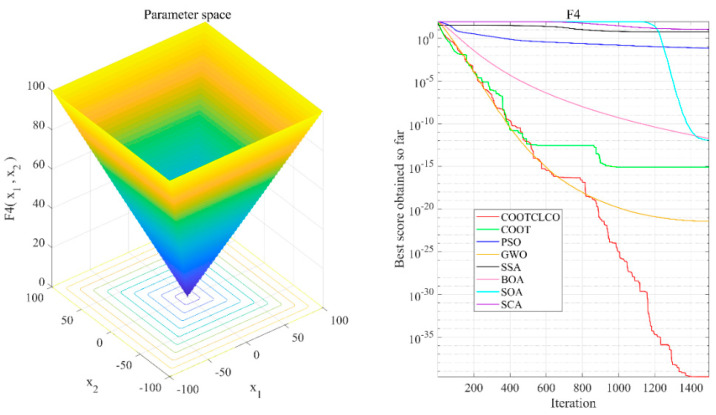
Test function F4 and comparison of convergence curves on F4.

**Figure 8:**
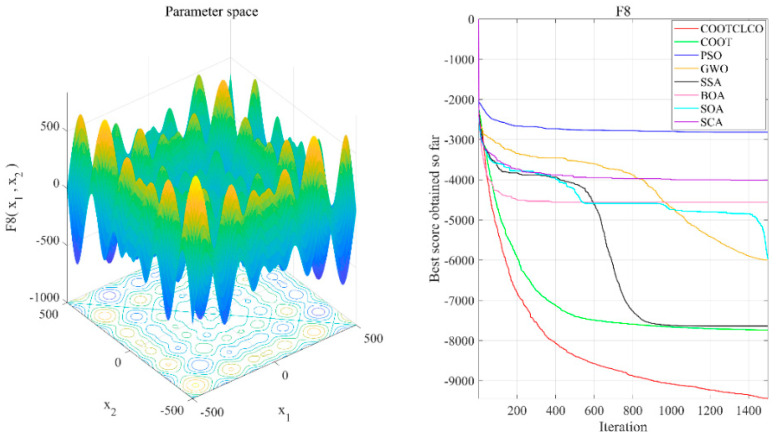
Test function F8 and comparison of convergence curves on F8.

**Figure 9:**
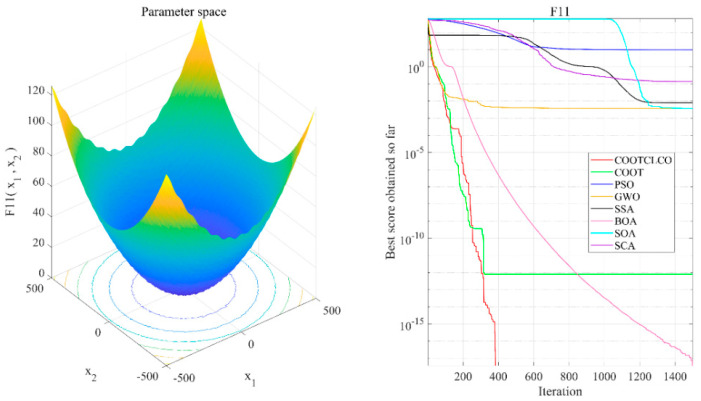
Test function F11 and comparison of convergence curves on F11.

**Figure 10:**
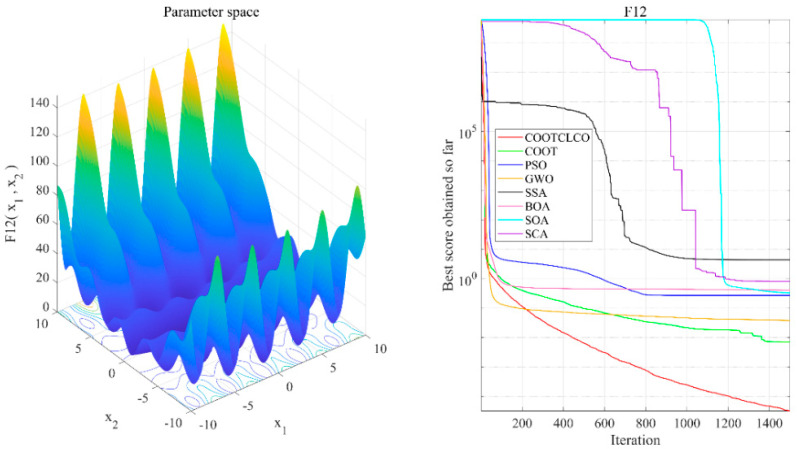
Test function F12 and comparison of convergence curves on F12.

**Figure 11:**
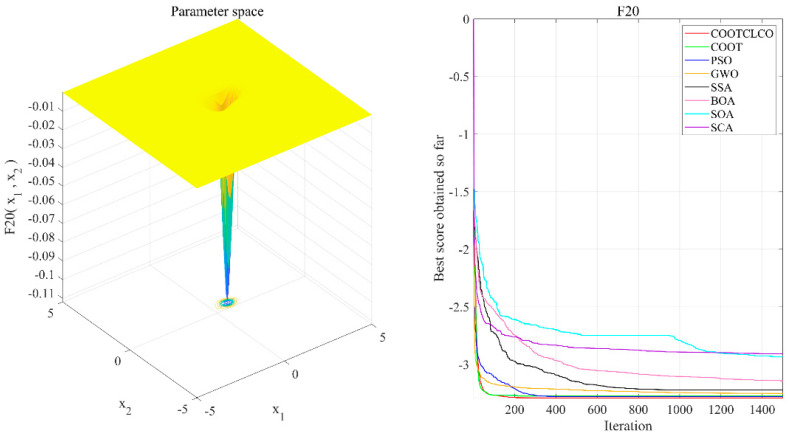
Test function F20 and comparison of convergence curves on F20.

**Figure 12:**
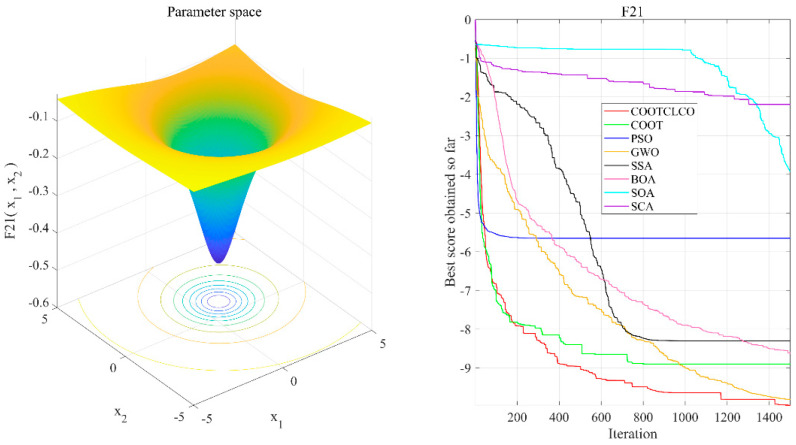
Test function F21 and comparison of convergence curves on F21.

Figure 6, Figure 7, Figure 8, Figure 9, Figure 10, Figure 11 and Figure 12 show the convergence curves of the eight algorithms of COOTCLCO, COOT, PSO, GWO, SSA, BOA, SOA, and SCA in the seven selected benchmark functions F3, F4, F8, F11, F12, F20, and F21, as well as the three-dimensional space diagram of the seven test functions. For all algorithms, the population size N=30 and the maximum number of iterations max_Iter=1500 are set. In order to reduce the contingency of the experiment, the curves given in these figures are the average convergence curves obtained from 30 independent experiments.

Figure 6 illustrates that COOTCLCO has a strong global search capability, and it finds a better global optimal value compared to other algorithms. It can quickly jump out of the local optimum in the middle stage of the algorithm’s iteration so as to continue to find and approach the theoretical optimal value of the function, which benefits from the effect of the Lévy flight mechanism and the algorithm’s improvement by combining the Cauchy mutation and the opposition-based learning. Although the search principle of COOT itself also has the ability to jump out of the local optimum, as can be seen from the figure, this ability is still not powerful enough compared to COOTCLCO. Figure 7 shows that the convergence rates of COOTCLCO, COOT, and GWO are very close in the early stages of the iteration, but both COOT and GWO fall into the local optimum too early, while COOTCLCO continues its search with a faster convergence rate until it obtains an optimal solution close to the theoretical optimal value. Figure 8 shows that PSO, SCA, and BOA fall into a stagnant state prematurely at the early stage of the algorithm’s iteration and, thus, produce bad search results. In contrast, COOTCLCO and COOT keep searching forward in a very stable state, and it can be seen from the figure that the whole process almost never falls into a local optimum, but COOTCLCO eventually has better search accuracy than COOT. Figure 9 shows that COOTCLCO was looking for the optimal value of the target with a very fast convergence speed, and eventually both COOTCLCO and BOA found results close to the theoretical optimal value. However, COOT was stuck in the local optimal state for most of the algorithm’s iteration, and performed poorly overall. Figure 10 shows that SOA, SCA, and SSA were at a standstill at the beginning of the algorithms’ iteration, and although the search started at a very fast convergence rate at the middle stage of the iteration, the algorithms fell into local optima at a later stage. Figure 11 shows that there is almost no difference in the convergence speed and convergence accuracy of the COOTCLCO, COOT, PSO, and GWO algorithms but, relatively speaking, the result of COOTCLCO is closest to the theoretical optimal value. Figure 12 shows that both COOTCLCO and GWO exhibit good search accuracy, but the convergence speed of COOTCLCO is significantly better than that of GWO. In summary, as the test results in Table 5, Table 6 and Table 7 and Figure 6, Figure 7, Figure 8, Figure 9, Figure 10, Figure 11 and Figure 12 show, COOTCLCO achieves very competitive results for most of the benchmark functions, indicating that it has reliable convergence speed as well as better exploration capability.

### 6.3. Coverage Performance Simulation Experiment and Analysis

To verify the effect of COOTCLCO on WSN node coverage optimization, six optimization algorithms—PSO, BOA, SOA, WOA, HHO, and BES—were selected for comparison on the WSN node coverage optimization problem. The sensor nodes were deployed in a square monitoring area of 100 m × 100 m, the sensing radius of the sensor nodes was *R_s_* = 10 m, the communication radius was *R_c_* = 20 m, the number of sensor nodes was denoted by *q*, and the number of iterations was denoted by *Iteration*. The experimental parameters of the node deployment area were set as shown in Table 9. Three sets of comparison experiments were designed in this section: (1) 30 independent experiments with different algorithms for 25, 35, and 45 sensor nodes, to plot their average coverage rate curves; (2) initial coverage diagram and coverage optimization diagram of COOTCLCO for node coverage optimization plotted for 25, 35, and 45 sensor nodes; (3) initial coverage diagram and coverage optimization diagram of COOTCLCO for node coverage optimization plotted for 45 sensor nodes and 500, 1000, and 1500 iterations.

#### 6.3.1. Comparative Experiment 1 and Result Analysis

**Figure 13:**
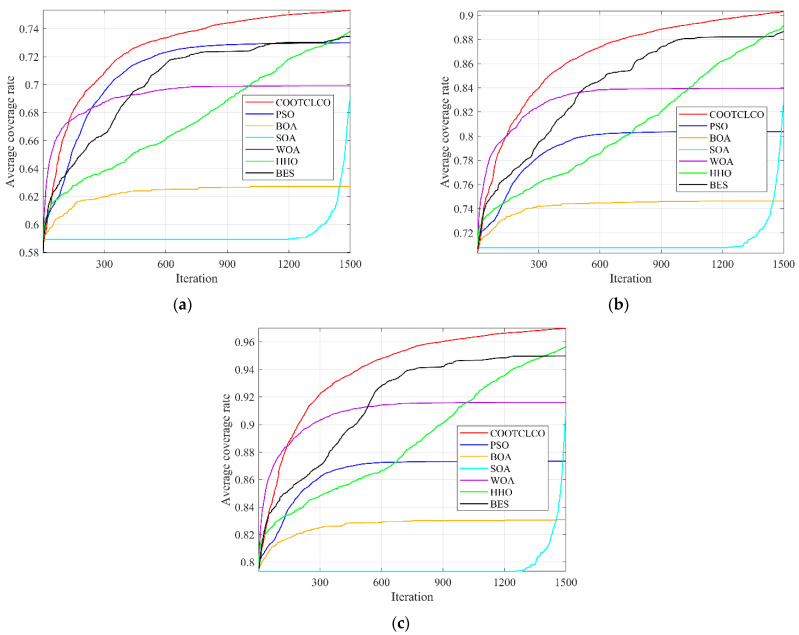
Comparison of the average coverage rate curves of the seven algorithms in the three cases: (**a**) *q* = 25, *Rs* = 10 m, *Iteration* = 1500; (**b**) *q* = 35, *Rs* = 10 m, *Iteration* = 1500; (**c**) *q* = 45, *Rs* = 10 m, *Iteration* = 1500.

**Figure 14:**
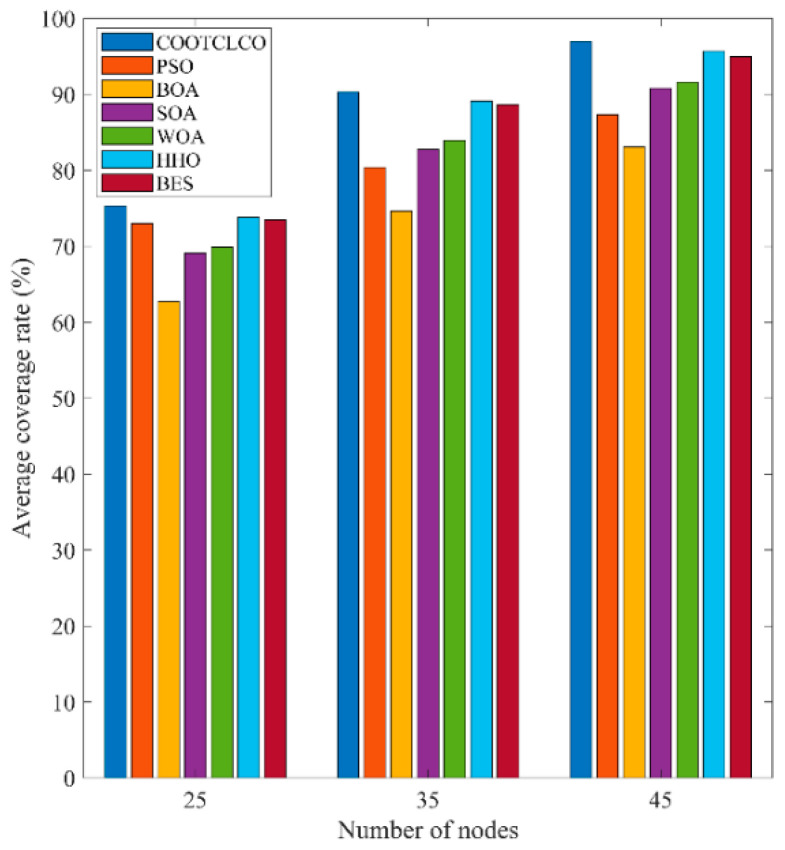
Comparison of average coverage rates under different numbers of nodes.

In the first type of comparative experiments, in order to reduce the contingency of the experiment, 30 independent experiments were conducted with different algorithms for 25, 35, and 45 sensor nodes; the average value of their coverage rate was taken, and their average coverage rate curves were plotted as shown in Figure 13. Table 10 gives the comparison of the average coverage rate results of the seven algorithms, and the comparison of the average coverage rates in the cases of different node numbers is shown in Figure 14. As can be seen from Figure 13, COOTCLCO achieved the best coverage rate in all three cases, and its average final coverage rate was 75.329%, 90.332% and 96.990%, respectively; of course, HHO and BES also obtained a good coverage effect, while BOA had the worst performance in node coverage optimization. In the case of changing only the number of sensor nodes, the coverage rate increased as the number of nodes continued to increase. Overall, COOTCLCO outperformed the other six algorithms in terms of WSN coverage optimization.

#### 6.3.2. Comparative Experiment 2 and Result Analysis

**Figure 15:**
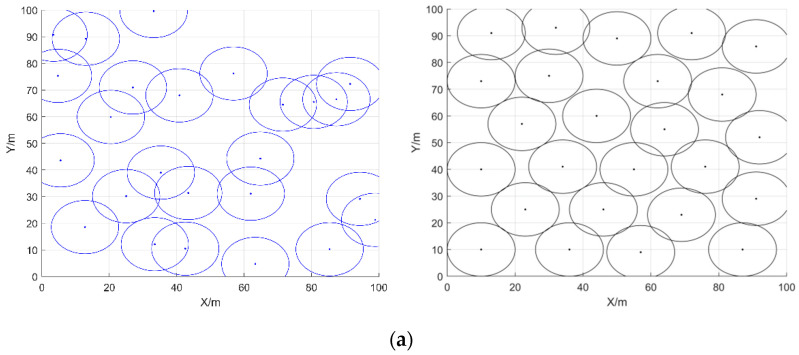

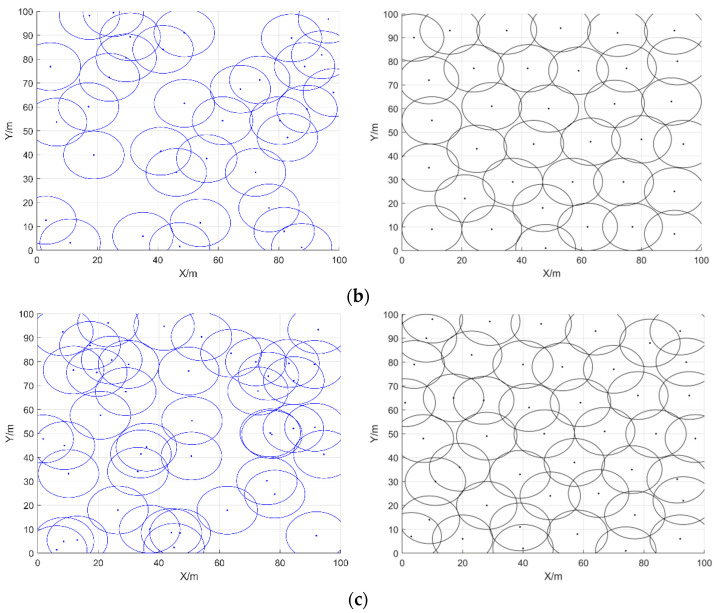
Initial coverage diagram and coverage optimization diagram of COOTCLCO in three cases: (**a**) *q* = 25, *Rs* = 10 m, *Iteration* = 1500; (**b**) *q* = 35, *Rs* = 10 m, *Iteration* = 1500; (**c**) *q* = 45, *Rs* = 10 m, *Iteration* = 1500.

**Figure 16:**
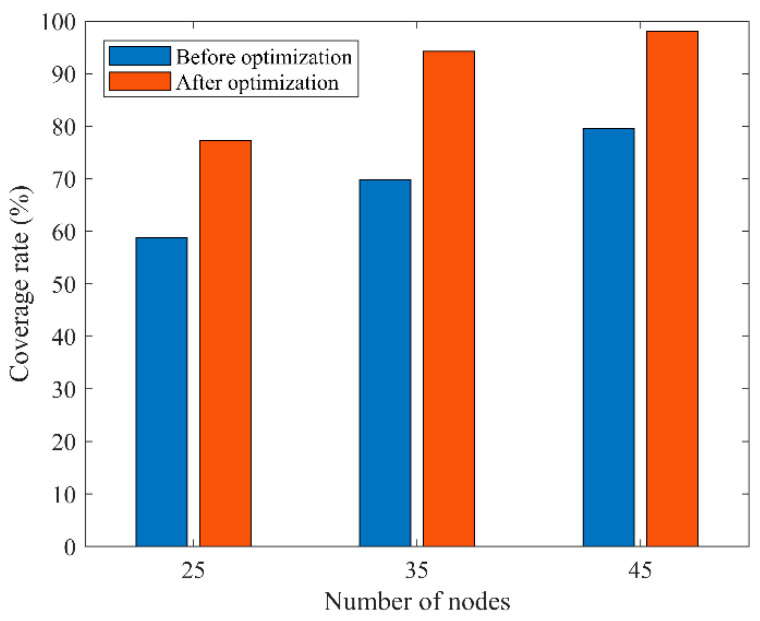
Comparison of coverage rates before and after optimization by COOTCLCO under different numbers of nodes.

In the second type of comparison experiments, different numbers of sensor nodes were randomly deployed in a 100 m × 100 m area with a sensing radius of 10 m and a communication radius of 20 m for each sensor node, and the maximum number of iterations was 1500. The initial coverage diagram and coverage optimization diagram of COOTCLCO for node coverage optimization are shown in Figure 15 for the cases of 25, 35, and 45 sensor nodes. Figure 16 gives a comparison of the coverage rates before and after optimization by COOTCLCO under different numbers of nodes. In Figure 15a, the number of sensor nodes is 25, and the initial coverage ratio of the coverage diagram initialized randomly is 58.81%, while after optimization by COOTCLCO the final coverage rate is obtained as 77.28%, which is an 18.47% increase in coverage. In Figure 15b, the number of sensor nodes is 35, and the initial coverage rate of the coverage diagram initialized randomly is 69.76%, while after optimization by COOTCLCO the final coverage rate is obtained as 94.23%, which is a 24.47% increase in coverage. In Figure 15c, the number of sensor nodes is 45, and the initial coverage rate of the coverage diagram initialized randomly is 79.61%, while after optimization by COOTCLCO the final coverage rate is obtained as 98.07%, which is an 18.46% increase in coverage. It can be seen that in these three cases, after COOTCLCO optimization, the locations of the randomly initialized sensor nodes become neat and orderly rather than haphazard, which effectively improves the coverage rate of the deployment area.

#### 6.3.3. Comparative Experiment 3 and Result Analysis

**Figure 17:**
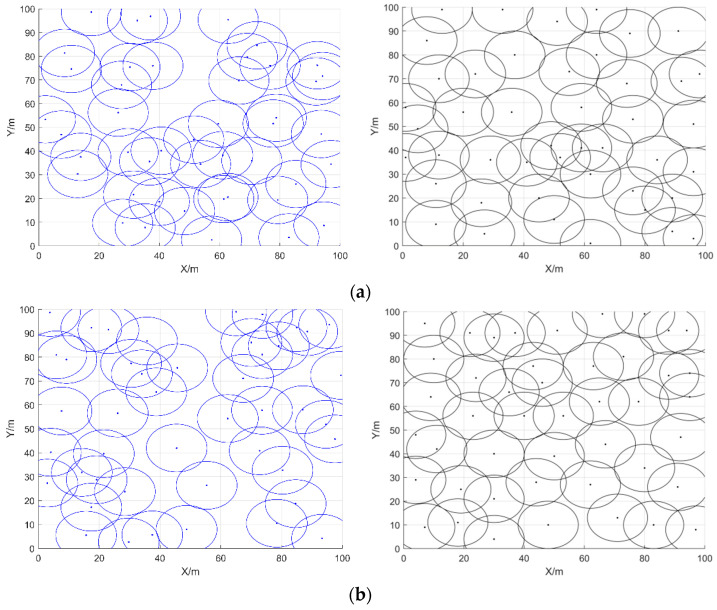

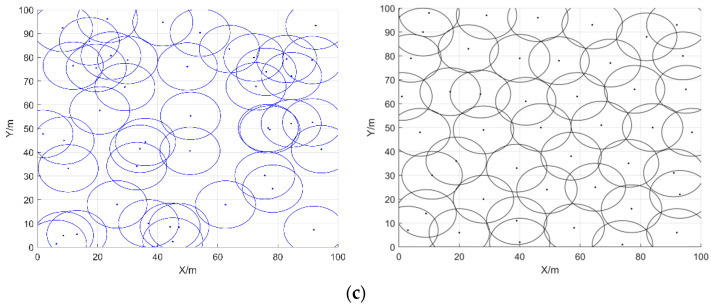
Initial coverage diagram and coverage optimization diagram of COOTCLCO in three cases: (**a**) *q* = 45, *Rs* = 10 m, *Iteration* = 500; (**b**) *q* = 45, *Rs* = 10 m, *Iteration* = 1000; (**c**) *q* = 45, *Rs* = 10 m, *Iteration* = 1500.

**Figure 18:**
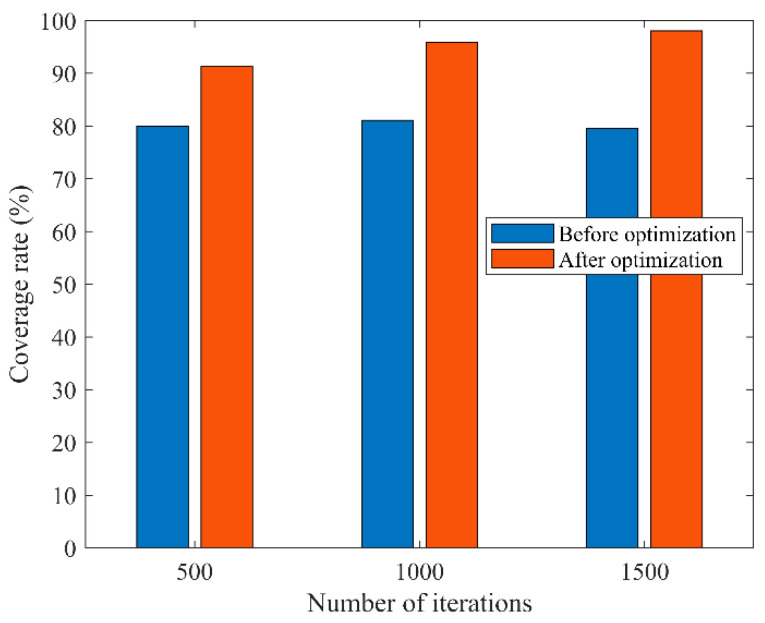
Comparison of coverage rates before and after optimization by COOTCLCO when the number of nodes is 45.

In the third type of comparison experiments, similarly, the area was set to 100 m × 100 m, the sensing radius of each sensor node was 10 m, and the communication radius was 20 m. We only changed the number of iterations, where the number of sensor nodes was 45 and the number of iterations was 500, 1000, or 1500; the initial coverage diagram of the nodes and the coverage diagram optimized by COOTCLCO are given in Figure 17. Figure 18 gives a comparison of the coverage rates before and after optimization by COOTCLCO when the number of nodes is 45. In Figure 17a, the number of iterations is set to 500, and the initial coverage rate of the coverage diagram initialized randomly is 79.95%, while after optimization by COOTCLCO the final coverage is obtained as 91.28%, which is an 11.33% increase in coverage. In Figure 17b, the number of iterations is set to 1000, and the initial coverage rate of the coverage diagram initialized randomly is 81.08%, while after optimization by COOTCLCO the final coverage is obtained as 95.86%, which is a 14.78% increase in coverage. In Figure 17c, the number of iterations is set to 1500, and the initial coverage rate of the coverage diagram initialized randomly is 79.61%, while after optimization by COOTCLCO the final coverage is obtained as 98.07%, which is an 18.46% increase in coverage. It can be seen that the final coverage rate increased by only 6.79% when the number of iterations was increased from 500 to 1500, while in the second group of comparison experiments, the final coverage rate improved by 20.79% when the number of sensor nodes was increased from 25 to 45. Therefore, in the case of changing only the number of sensor nodes or only the number of iterations, reasonably and finitely increasing the number of sensor nodes is more likely to greatly improve the coverage rate of the target monitoring area compared to increasing the number of iterations.

## 7. Conclusions

Aiming at the problems of uneven node distribution and low coverage of the target monitoring area when randomly deploying sensor nodes in WSNs, a COOTCLCO algorithm for node coverage optimization in WSNs is proposed in the paper. COOTCLCO uses a chaotic tent map to initialize the population based on the original COOT algorithm, which increases the diversity of the population and enhances the traversal of the search space by the COOT population. The Lévy flight strategy is introduced to perturb individual positions, which can expand the search range of the population and reduce the possibility of the algorithm falling into a local optimum. The algorithm then combines the Cauchy mutation and the opposition-based learning strategy to perturb the optimal solution positions and generate new solutions, which further enhances the ability of the algorithm to jump out of the local optimum. In order to verify the optimization performance of COOTCLCO, 23 benchmark functions were used to test the optimization performance of the algorithm, which was compared with seven other optimization algorithms: COOT, PSO, GWO, SSA, BOA, SOA and SCA. By analyzing the numerical results and convergence curves of the simulation experiments, we found that COOTCLCO has reliable convergence speed as well as better global exploration capability. To verify the capability of COOTCLCO on the WSN node coverage optimization problem, we compared it with six optimization algorithms, namely, PSO, BOA, SOA, WOA, HHO, and BES. The experimental results show that COOTCLCO obtained the highest average coverage rate under the same test conditions, and the coverage rate convergence curves indicate that COOTCLCO can improve the coverage rate of WSN nodes quickly and effectively. This means that COOTCLCO only requires the least number of sensor nodes to achieve the same coverage rate in the same target monitoring area, which reduces the deployment cost of sensor nodes.

However, the work of this paper has some limitations. Although our proposed algorithm has a significant effect on the improvement of the coverage rate, there are still shortcomings in the experimental design. Firstly, our experiments were designed to optimize the coverage rate in an ideal environment, while in a real complex environment the influence of obstacles should be taken into account. Secondly, this paper only considers the coverage optimization of a two-dimensional plane, and the coverage optimization of wireless sensor networks can be extended to three-dimensional space. Thirdly, the coverage optimization objective of this paper is relatively singular, and only the coverage rate optimization is considered, while the other coverage optimization indices of wireless sensor networks such as network energy consumption, network life cycle, network security, and sensor power optimization should also be taken into consideration in the real environment. Therefore, for the next step, we will continue to improve and refine the algorithm and conduct further research on the above-mentioned limitations.

## Figures and Tables

**Table 1:** Notation descriptions.

Notations	Descriptions
*q*	Number of sensor nodes
*n*	Number of target monitoring points
*M* × *N*	Size of the monitoring area
*S_i_*	The *i*-th sensor node
*x_i_*, *y_i_*	The location coordinates of each sensor node
*T_j_*	The *j*-th target monitoring point
*x_j_*, *y_j_*	The location coordinates of each target point
*R_s_*	Sensing radius
*R_c_*	Communication radius
*d*(*S_i_*, *T_j_*)	The Euclidean distance between *S_i_* and *T_j_*
*p*	Probability of monitoring points being covered by nodes
*P*	Probability of monitoring points being jointly sensed
*Cov*	Coverage rate
*CootPos*(*i*)	The position of the *i*-th COOT
*LeaderPos*(*k*)	The position of the selected leader
*d*	The number of variables or problem dimensions
*ub*	The upper bound of the search space
*lb*	The lower bound of the search space
*Q*	Random initialization of the location
*A*, *B*	Control parameters
*NL*	The number of leaders
*R_1_*, *R_2_*, *R_3_*, *R_4_*	The random numbers between the interval [0, 1]
*R*	The random number between the interval [−1, 1]
*α*, *µ*, *γ*, *λ*, *v*	Control parameters
*s*	Search path of the Lévy flight
*X*’*_LeaderPos_*_(*i*)_	The inverse solution of the current leader position
*max_Iter*	Maximum number of iterations
*X_GbestNew_*	The latest position after perturbed by Cauchy mutation
*P_s_*	The selection probability

**Table 2:** Unimodal benchmark functions.

F	Function	Dim	Range	*f*_min_
F1	$f_{1}(x)=\overset{n}{\underset{i=1}{\sum }}x_{i}^{2}$	30	[−100, 100]	0
F2	$f_{2}(x)=\overset{n}{\underset{i=1}{\sum }}\left|x_{i}\right|+\overset{n}{\underset{i=1}{\prod }}\left|x_{i}\right|$	30	[−10, 10]	0
F3	$f_{3}(x)=\overset{n}{\underset{i=1}{\sum }}{\left(\overset{i}{\underset{j-1}{\sum }}x_{j}\right)}^{2}$	30	[−100, 100]	0
F4	$f_{4}(x)=\max\left\{\left|x_{i}\right|,1⩽i⩽n\right\}$	30	[−100, 100]	0
F5	$f_{5}(x)=\overset{n-1}{\underset{i=1}{\sum }}\left[100{\left(x_{i+1}-x_{i}^{2}\right)}^{2}+{\left(x_{i}-1\right)}^{2}\right]$	30	[−30, 30]	0
F6	$f_{6}(x)=\overset{n}{\underset{i=1}{\sum }}{\left(\left[x_{i}+0.5\right]\right)}^{2}$	30	[−100, 100]	0
F7	$f_{7}(x)=\max\left\{\left|x_{i}\right|,1⩽i⩽n\right\}$	30	[−1.28, 1.28]	0

**Table 3:** Multimodal benchmark functions.

F	Function	Dim	Range	*f*_min_
F8	$f_{8}(x)=\overset{n}{\underset{i=1}{\sum }}-x_{i}\sin\left(\sqrt{\left|x_{i}\right|}\right)$	30	[−500, 500]	−418.9829×n
F9	$f_{9}(x)=\overset{n}{\underset{i=1}{\sum }}x_{i}^{2}-10\cos\left(2\pi x_{i}\right)+10$	30	[−5.12, 5.12]	0
F10	$\begin{array}{l} f_{10}(x)=-20\exp\left(-0.2\sqrt{\frac{1}{n}\overset{n}{\underset{i=1}{\sum }}x_{i}^{2}}\right)-\\ \exp\left(\frac{1}{n}\overset{n}{\underset{i}{\sum }}\cos\left(2\pi x_{i}\right)\right)+20+e \end{array}$	30	[−32, 32]	0
F11	$f_{11}(x)=\overset{n}{\underset{i=1}{\sum }}\frac{x_{i}^{2}}{4000}-\overset{n}{\underset{i=1}{\prod }}\cos\left(\frac{x_{i}}{\sqrt{i}}\right)-1$	30	[−600, 600]	0
F12	$\begin{array}{l} f_{12}(x)=\frac{\pi }{n}\left\{\begin{array}{l} 10\sin^{2}\left(\pi y_{1}\right)+\overset{n-1}{\underset{i=1}{\sum }}{\left(y_{i}-1\right)}^{2}\\ \left[1+10\sin^{2}\left(\pi y_{i+1}\right)\right]+{\left(y_{n}-1\right)}^{2} \end{array}\right\}\\ +\overset{n}{\underset{i=1}{\sum }}u\left(x_{i},10,100,4\right)\\ y_{i}=1+\frac{x_{i}+1}{4},\;\\ u\left(x_{i},a,k,m\right)=\left\{\begin{array}{c} k{\left(x_{i}-a\right)}^{m}\;x_{i}>a\\ 0\;-a<x_{i}<a\\ k{\left(-x_{i}-a\right)}^{m}x_{i}<-a \end{array}\right\} \end{array}$	30	[−50, 50]	0
F13	$\begin{array}{l} f_{13}(x)=0.1\left\{\begin{array}{l} \begin{array}{l} \sin^{2}\left(3\pi x_{i}\right)+\overset{n}{\underset{i=1}{\sum }}{\left(x_{i}-1\right)}^{2}\\ \left[1+\sin^{2}\left(3\pi x_{i}+1\right)\right] \end{array}\\ +{\left(x_{n}-1\right)}^{2}\left[1+\sin^{2}\left(2\pi x_{n}\right)\right] \end{array}\right\}\\ +\overset{n}{\underset{i=1}{\sum }}u\left(x_{i},5,100,4\right) \end{array}$	30	[−50, 50]	0

**Table 4:** Fixed-dimension multimodal benchmark functions.

F	Function	Dim	Range	*f*_min_
F14	$f_{14}(x)={\left(\frac{1}{500}+\overset{25}{\underset{j=1}{\sum }}\frac{1}{j+\sum_{i=1}^{2}{\left(x_{i}-a_{ij}\right)}^{6}}\right)}^{-1}$	2	[−65, 65]	1
F15	$f_{15}(x)=\overset{11}{\underset{i=1}{\sum }}{\left[a_{i}-\frac{x_{1}\left(b_{i}^{2}+b_{i}x_{2}\right)}{b_{i}^{2}+b_{i}x_{3}+x_{4}}\right]}^{2}$	4	[−5, 5]	0.0003
F16	$f_{16}(x)=4x_{1}^{2}-2.1x_{1}^{4}+\frac{1}{3}x_{1}^{6}+x_{1}x_{2}-4x_{2}^{2}+4x_{2}^{4}$	2	[−5, 5]	−1.0316
F17	$\begin{array}{l} f_{17}(x)={\left(x_{2}-\frac{5.1}{4\pi^{2}}x_{1}^{2}+\frac{5}{\pi }x_{1}-6\right)}^{2}\\ +10\left(1-\frac{1}{8\pi }\right)\cos x_{1}+10 \end{array}$	2	[−5, 5]	0.398
F18	$\begin{array}{l} f_{18}(x)=\left[1+{\left(x_{1}+x_{2}+1\right)}^{2}\left(\begin{array}{l} 19-14x_{1}+\\ 3x_{1}^{2}-14x_{2}+\\ 6x_{1}x_{2}+3x_{2}^{2} \end{array}\right)\right]\\ \times \left[30+{\left(2x_{1}-3x_{2}\right)}^{2}\times \left(\begin{array}{l} 18-32x_{1}+\\ 12x_{1}^{2}+48x_{2}-\\ 36x_{1}x_{2}+27x_{2}^{2} \end{array}\right)\right] \end{array}$	2	[−2, 2]	3
F19	$f_{19}(x)=-\overset{4}{\underset{i=1}{\sum }}c_{i}\exp\left(-\overset{3}{\underset{j=1}{\sum }}a_{ij}{\left(x_{j}-p_{ij}\right)}^{2}\right)$	3	[1, 3]	−3.86
F20	$f_{20}(x)=-\overset{4}{\underset{i=1}{\sum }}c_{i}\exp\left(-\overset{6}{\underset{j=1}{\sum }}a_{ij}{\left(x_{j}-p_{ij}\right)}^{2}\right)$	6	[0, 1]	−3.32
F21	$f_{21}(x)=-\overset{5}{\underset{i=1}{\sum }}{\left[\left(X-a_{i}\right){\left(X-a_{i}\right)}^{T}+c_{i}\right]}^{-1}$	4	[0, 10]	−10.1532
F22	$f_{22}(x)=-\overset{7}{\underset{i=1}{\sum }}{\left[\left(X-a_{i}\right){\left(X-a_{i}\right)}^{T}+c_{i}\right]}^{-1}$	4	[0, 10]	−10.4028
F23	$f_{23}(x)=-\overset{10}{\underset{i=1}{\sum }}{\left[\left(X-a_{i}\right){\left(X-a_{i}\right)}^{T}+c_{i}\right]}^{-1}$	4	[0, 10]	−10.5363

**Table 5:** Parameter settings of the algorithm.

Algorithms	Parameters	Values
COOTCLCO	Population	30
	Iteration	1500
	*R*	[−1, 1]
	*R*_1_	[0, 1]
	*R*_2_	[0, 1]
	*µ*	2
	*r*	*tan*((*rand*( )−0.5) × 0.5)
COOT	Population	30
	Iteration	1500
	*R*	[−1, 1]
	*R*_1_	[0, 1]
	*R*_2_	[0, 1]
PSO	Population	30
	Iteration	1500
	*c*_1_, *c*_2_	2
	*w*_min_	0.2
	*w*_max_	0.9
GWO	Population	30
	Iteration	1500
	*a*	[2, 0]
SSA	Population	30
	Iteration	1500
	*c*_1_, *c*_2_, *c*_3_	[0, 1]
BOA	Population	30
	Iteration	1500
	*a*	0.1
	*c*	0.01
	*p*	0.6
SOA	Population	30
	Iteration	1500
	*A*	[2, 0]
	*f*_c_	2
SCA	Population	30
	Iteration	1500
	*a*	2
	*r*_1_, *r*_2_, *r*_3_, *r*_4_	[0, 1]

**Table 6:** Results of unimodal and multimodal benchmark functions.

Function	Criteria	COOTCLCO	COOT	PSO	GWO	SSA	BOA	SOA	SCA
F1	avg	2.659 × 10^−83^	1.0898 × 10^−31^	4.6753 × 10^−13^	**2.3081 × 10^−90^**	8.514 × 10^−09^	2.4291 × 10^−15^	6.4176 × 10^−43^	0.00021788
	std	1.4561 × 10^−82^	5.9691 × 10^−31^	1.5343 × 10^−12^	**9.858 × 10^−90^**	1.546 × 10^−09^	1.6651 × 10^−16^	1.831 × 10^−42^	0.0010654
	W	/	+	+	≈	+	+	+	+
	R	2	4	6	1	7	5	3	8
F2	avg	6.2512 × 10^−36^	6.4608 × 10^−21^	1.2222 × 10^−05^	**1.4253 × 10^−52^**	0.81098	1.6096 × 10^−12^	3.8153 × 10^−27^	2.9312 × 10^−08^
	std	3.3024 × 10^−35^	3.5387 × 10^−20^	3.694 × 10^−05^	**2.1289 × 10^−52^**	1.0753	1.3002 × 10^−13^	9.4533 × 10^−27^	6.6456 × 10^−08^
	W	/	+	+	−	+	+	≈	+
	R	2	4	7	1	8	5	3	6
F3	avg	**3.5233 × 10^−77^**	6.9729 × 10^−38^	0.19834	3.1175 × 10^−23^	30.5211	2.09 × 10^−15^	6.7979 × 10^−23^	2114.2643
	std	**1.9298 × 10^−76^**	3.8192 × 10^−37^	0.10306	1.573 × 10^−22^	25.2264	1.5103 × 10^−16^	1.5973 × 10^−22^	2264.7997
	W	/	+	+	+	+	+	+	+
	R	1	2	6	4	7	5	3	8
F4	avg	**1.7579 × 10^−29^**	5.3006 × 10^−22^	0.065309	3.1451 × 10^−22^	5.0517	1.8376 × 10^−12^	1.4693 × 10^−13^	10.2213
	std	**9.6209 × 10^−29^**	2.8788 × 10^−21^	0.026898	4.7313 × 10^−22^	2.7223	1.1907 × 10^−13^	3.593 × 10^−13^	8.0463
	W	/	≈	+	≈	+	+	+	+
	R	1	2	6	3	7	5	4	8
F5	avg	27.8032	49.0883	30.6025	**26.5966**	160.312	28.9041	27.9173	40.7895
	std	0.22848	64.9583	17.6035	**0.91726**	312.6524	0.025756	0.73362	50.9841
	W	/	+	+	−	+	+	≈	+
	R	2	7	5	1	8	4	3	6
F6	avg	0.0027052	0.0011474	**8.5311 × 10^−13^**	0.64475	9.294 × 10^−09^	5.0655	3.2155	4.3367
	std	0.001689	0.0006967	**3.7915 × 10^−12^**	0.34548	2.227 × 10^−09^	0.63497	0.44021	0.41554
	W	/	−	−	+	−	+	+	+
	R	4	3	1	5	2	8	6	7
F7	avg	0.0012201	0.0016025	0.029791	**0.00052173**	0.06215	0.00063614	0.00071636	0.018585
	std	0.0010915	0.0012723	0.0095664	**0.00031804**	0.026099	0.00021572	0.00056061	0.016281
	W	/	≈	+	−	+	−	−	+
	R	4	5	7	1	8	2	3	6
F8	avg	**−9259.1292**	−7727.7499	−2947.3405	−6119.6079	−7766.648	−4520.5507	−5435.957	−3978.2828
	std	**746.799**	843.0888	528.7386	453.4792	689.3375	324.7176	662.0541	259.1258
	W	/	+	+	+	+	+	+	+
	R	1	3	8	4	2	6	5	7
F9	avg	**5.6843 × 10^−15^**	4.3428 × 10^−12^	47.8575	0.38537	57.9397	29.8787	2.0138	11.9765
	std	**1.7345 × 10^−14^**	2.3765 × 10^−11^	14.9651	1.4669	16.6952	68.3273	7.7314	21.3326
	W	/	≈	+	+	+	+	+	+
	R	1	2	7	3	8	6	4	5
F10	avg	**4.1034 × 10^−14^**	1.2819 × 10^−13^	9.0532 × 10^−08^	1.1191 × 10^−14^	1.8622	5.2254 × 10^−13^	19.9593	16.2366
	std	**1.5409 × 10^−13^**	3.9915 × 10^−13^	1.7522 × 10^−07^	3.2788 × 10^−15^	0.87668	3.8682 × 10^−13^	0.0012739	7.4322
	W	/	≈	+	≈	+	≈	+	+
	R	1	4	5	2	6	3	8	7
F11	avg	3.7007 × 10^−17^	4.9516 × 10^−15^	9.4859	0.0018945	0.010581	**3.7007 × 10^−18^**	0.002286	0.26667
	std	8.9073 × 10^−17^	2.6891 × 10^−14^	3.9401	0.0051435	0.010628	**2.027 × 10^−17^**	0.0092326	0.27876
	W	/	+	+	+	+	−	+	+
	R	2	3	8	4	6	1	5	7
F12	avg	**2.5932 × 10^−05^**	0.070101	0.34582	0.038975	5.4261	0.40324	0.2896	1.1145
	std	**1.7952 × 10^−05^**	0.21764	0.50576	0.020464	4.5121	0.14044	0.1326	1.0823
	W	/	+	+	+	+	+	+	+
	R	1	3	5	2	8	6	4	7
F13	avg	0.0085188	0.015734	**0.00036625**	0.45024	0.5924	2.4769	1.9698	24.3547
	std	0.011135	0.023316	**0.002006**	0.2401	3.2058	0.38948	0.15777	104.6906
	W	/	+	−	+	+	+	+	+
	R	2	3	1	4	5	7	6	8
+/≈/−		/	8/4/1	11/2/0	7/3/3	12/0/1	10/1/2	10/2/1	13/0/0

**Table 7:** Results of fixed-dimension multimodal benchmark functions.

Function	Criteria	COOTCLCO	COOT	PSO	GWO	SSA	BOA	SOA	SCA
F14	avg	**0.998**	0.998	1.6906	4.1922	0.998	1.0643	1.3948	1.3287
	std	**3.0018** **×** **10^−16^**	2.2395 × 10^−16^	1.4911	4.4183	1.725 × 10^−16^	0.36262	0.80721	0.75206
	W	/	≈	+	+	≈	+	+	+
	R	1	2	7	8	3	4	6	5
F15	avg	0.00046961	0.00064839	0.0004961	0.005088	0.00082478	**0.00032609**	0.0011046	0.00081571
	std	0.00020712	0.00031895	0.00039601	0.0085754	0.00024494	**1.7582** **×** **10^−05^**	0.00031781	0.00030811
	W	/	+	≈	+	+	−	+	+
	R	2	4	3	8	6	1	7	5
F16	avg	**−1.0316**	−1.0316	−1.0316	−1.0316	−1.0316	−1.0316	−1.0316	−1.0316
	std	**6.8377** **×** **10^−16^**	1.8251 × 10^−12^	6.7752 × 10^−16^	2.3368 × 10^−09^	3.931 × 10^−15^	1.0339 × 10^−05^	1.4916 × 10^−07^	1.4874 × 10^−05^
	W	/	≈	≈	≈	≈	+	+	+
	R	1	4	2	5	3	8	6	7
F17	avg	**0.39789**	0.39789	**0.39789**	0.39789	0.39789	0.39823	0.3979	0.39841
	std	**0**	1.2974 × 10^−15^	**0**	7.2275 × 10^−08^	2.070 × 10^−15^	0.00079194	1.0205 × 10^−05^	0.00052896
	W	/	+	≈	+	≈	+	+	+
	R	1	4	1	5	3	7	6	8
F18	avg	**3**	3	3	3	3	3.0112	3	3
	std	**3.2769** **×** **10^−15^**	1.5417 × 10^−14^	1.7916 × 10^−15^	7.3174 × 10^−06^	3.959 × 10^−14^	0.032131	1.9517 × 10^−05^	4.1425 × 10^−05^
	W	/	+	≈	+	≈	+	+	+
	R	1	4	2	5	3	8	7	6
F19	avg	−0.30048	−0.30048	**−3.8628**	−0.30048	−0.30048	−0.30048	−0.30048	−0.30048
	std	2.2584 × 10^−16^	2.2584 × 10^−16^	**2.7101** **×** **10^−15^**	2.2584 × 10^−16^	2.259 × 10^−16^	2.2584 × 10^−16^	2.2584 × 10^−16^	2.2584 × 10^−16^
	W	/	≈	−	+	≈	+	+	+
	R	2	3	1	7	4	6	5	8
F20	avg	**−3.2982**	−3.2943	−3.2625	−3.277	−3.2263	−3.1381	−2.7975	−2.8919
	std	**0.04837**	0.051146	0.060463	0.073544	0.048682	0.14729	0.54644	0.39914
	W	/	≈	+	+	+	+	+	+
	R	1	2	4	3	5	6	8	7
F21	avg	**−9.6924**	−9.2305	−6.3881	−9.1395	−7.5573	−9.0466	−3.1981	−3.0402
	std	**1.3421**	2.1365	3.4666	2.0617	3.122	0.94949	3.9074	2.2051
	W	/	+	+	+	+	+	+	+
	R	1	2	6	3	5	4	7	8
F22	avg	−9.5169	−10.2271	−6.578	**−10.4027**	−9.2863	−9.4478	−6.1925	−3.9181
	std	2.0147	0.96292	3.5147	**0.00013733**	2.584	1.1399	4.6827	1.9622
	W	/	−	+	−	+	≈	+	+
	R	3	2	6	1	5	4	7	8
F23	avg	−9.7655	−10.3577	−8.1972	**−10.5362**	−9.4872	−10.0631	−8.1032	−4.9455
	std	1.8698	0.97874	3.6466	**0.00010617**	2.4332	0.3434	3.9097	1.9771
	W	/	−	+	−	≈	−	+	+
	R	4	2	6	1	5	3	7	8
+/≈/−		/	4/4/2	5/4/1	7/1/2	4/6/0	7/1/2	10/0/0	10/0/0

**Table 8:** Overall Wilcoxon signed-rank test results, average value rank results, and algorithm rank results.

Result	COOTCLCO	COOT	PSO	GWO	SSA	BOA	SOA	SCA
+/≈/−	/	12/8/3	16/6/1	14/4/5	16/6/1	17/2/4	20/2/1	23/0/0
Average rank	1.783	3.087	4.696	3.522	5.391	4.957	5.348	6.957
Overall rank	1	2	4	3	7	5	6	8

**Table 9:** Experimental parameter settings for the node deployment area.

Parameters	Values
Area of deployment	100 m × 100 m
Sensing radius (*R_s_*)	10 m
Communication radius (*R_c_*)	20 m
Number of sensor nodes (*q*)	25, 35, 45
Number of iterations (*Iteration*)	500, 1000, 1500

**Table 10:** Comparison of average coverage rate.

Algorithm	*q* = 25	*q* = 35	*q* = 45
	Average Coverage Rate/%	Average Coverage Rate/%	Average Coverage Rate/%
COOTCLCO	75.329	90.332	96.990
PSO	72.999	80.383	87.336
BOA	62.718	74.634	83.102
SOA	69.066	82.752	90.802
WOA	69.913	83.947	91.600
HHO	73.831	89.115	95.680
BES	73.459	88.643	94.978

## Data Availability

Not applicable.
